# Green Synthesis of Silver Nanoparticles from *Camellia sinensis* and Its Antimicrobial and Antibiofilm Effect against Clinical Isolates

**DOI:** 10.3390/ma15196978

**Published:** 2022-10-08

**Authors:** Syed Ghazanfar Ali, Mohammad Jalal, Hilal Ahmad, Diwakar Sharma, Akil Ahmad, Khalid Umar, Haris Manzoor Khan

**Affiliations:** 1 Department of Microbiology, Jawaharlal Nehru Medical College, Aligarh Muslim University, Aligarh 202002, India; 2 SRM Institute of Science and Technology, Kattankulathur, Chennai 603203, India; 3 Department of Civil Engineering, Aligarh Muslim University, Aligarh 202002, India; 4 Department of Chemistry, College of Science and Humanities in Al-Kharj, Prince Sattam Bin Abdulaziz University, Al-Kharj 11942, Saudi Arabia; 5 School of Chemical Sciences, University Sains Malaysia, Gelugor 11800, Malaysia

**Keywords:** *Camellia sinensis*, CLSM, MIC, SEM, silver nanoparticles, TEM

## Abstract

The green synthesis method of was used for the synthesis of silver nanoparticles using *Camellia sinensis* (green tea). The *Camellia sinensis* silver nanoparticles (CS-AgNPs) were characterized using different techniques, including UV-Vis (ultra violet-visible), SEM (scanning electron microscopy), TEM (transmission electron microscopy), and XRD (X-ray diffraction). The average size of the CS-AgNPs was 52 nm, according to TEM. The CS-AgNPs showed excellent antibacterial and antifungal activity. The MIC (minimum inhibitory concentration) against bacterial isolates varied from 31.25 to 62.5 µg/mL, whereas for fungal isolates, the MIC varied from 125 to 250 µg/mL. The presence of a zone in the well diffusion assay showed the antimicrobial nature of CS-AgNPs. Further, CLSM (confocal laser scanning microscopy) showed that CS-AgNPs possess antibiofilm activity. The interaction of CS-AgNPs with the Candidal cells was analyzed using TEM, and it was revealed that CS-AgNPs entered the cell and disrupted the cell machinery.

## 1. Introduction

Nanotechnology is the study of material at a very small scale, typically at the nanoscale level [1,2,3,4]. The nanomaterials have unique properties which differ from their bulky materials [5]. The small size increases the biochemical and catalytic activity due to the change in the surface-to-volume ratio [6,7]. Among the metallic nanoparticles, the silver nanoparticles are exceptional and are the most explored of the nanoparticles because of their simplicity of synthesis, versatility, adaptability, morphology, and high surface area [8]. The other reasons for considering silver nanoparticles is their antimicrobial efficacy, with low toxicity, and higher application both in vivo and in vitro [9]. Silver nanoparticles, due to the high surface-to-volume ratio and presence of a greater number of surface atoms, possess enhanced antibacterial activity compared with pure silver metal [10]. However, the actual mechanism of interaction of silver nanoparticles with microorganisms is still unknown, but there are a few proposed mechanisms. The large surface area of nanoparticles allows for the easy penetration or attachment of nanoparticles to the microbial cell wall [11], which disturbs the cell`s permeability and makes it porous, leading to cell leakage [12]. The nanoparticles entering through the pores also lead to the inactivation of proteins and damage to the DNA, since they bind with sulfur and phosphorous-containing proteins [13]. Another mechanism suggests the release of Ag^+^, which reacts with the thiol group of different enzymes and proteins, finally interfering with the respiratory chain. The Ag^+^ ions are also responsible for the release of reactive oxygen species (ROS), which causes cell death by inactivation of DNA replication and interference with ATP production [14].

Several methods are available for synthesizing metal and metal oxide nanoparticles, which include physical, chemical, and green methods [15,16]. The green method of nanoparticle synthesis is non-toxic [17], pollution free [18], and cost effective [19]; therefore, it is preferred over physical and chemical methods. Moreover, it does not require complex equipment or special synthesis conditions [20,21]. The green method of nanoparticle synthesis includes the use of a plant or part of the plants, since plants are good sources of metabolites, which helps in reducing the metal salt, such as silver, and also help in the stabilizing and capping of nanoparticles [22]. The plant-based synthesis of nanoparticles is very simple, since it requires only metal salt and plant extract for the reduction [23]. The application of plant extract in the green synthesis of nanoparticles due to its reducing and stabilizing properties has attracted many researchers worldwide. The medicinal plants are rich source of biomolecules, phytochemicals, and antioxidants, such as polyphenols, carotenoids, polysaccharides, aldehydes, ketones, proteins, enzymes, amino acids, and caffeine [24]. These complex biomolecules assist in the reduction of metal ions and also provide stability to nanoparticles [13]. The green method of nanoparticle synthesis has been reported in different plants, including *Tithonia diversifolia* [25], *Acalypha wilkesiana* [26], *Holarrhena pubescens* [27] from the fern *Gleichenia pectinata* [28], and from different vegetable species [29].

Antibiotic resistance is becoming a serious health challenge worldwide, since the excessive use of antibiotic is allowing the microorganisms to develop resistance against antibiotics. It has become the most serious challenge regarding hospital, as well as community-acquired infections, more specifically caused due to the multi-drug resistant microorganisms [30]. Different studies have suggested the development of new microbial strains which possess antibiotic resistant genes [30]. Biofilm formation, which is an irreversible attachment of a colony of microorganisms on the surface, is another problem that has become difficult to eradicate. The exact mechanism of resistance is still unknown, but some factors, such as matrix polysaccharide protection [31] and efflux pump over-expression [32], may contribute to the resistance mechanism. Biofilm formation provides a suitable environment for stable colonization in host tissue, which further provides resistance to environmental stresses and resistance to antifungal and oxidative stress [33,34]. Therefore, an antimicrobial and antibiofilm alternative, which will not develop drug resistance, should be made available. 

Keeping in view the importance of plants and their beneficial role in synthesizing silver nanoparticles, we have synthesized silver nanoparticles using the extract of *Camellia sinensis* and analyzed their role as an antimicrobial and antibiofilm.

## 2. Material and Methods

### 2.1. Preparation of Camellia sinensis Extracts (CS)

*Camellia sinensis* (green tea) was purchased from the market and was sun dried and ground into powder form. Dried powder (10 g) was then mixed with the deionized water (100 mL) and kept in rotary shaker for 10 min at 60 rpm. The aqueous extract was then passed through the filter paper (Whatman No. 1, Merckmillipore), and the extract was stored at 4 °C for further use.

### 2.2. Synthesis of Camellia sinensis Silver Nanoparticles (CS-AgNPs)

10 mL of *Camellia sinensis* extract was mixed with the 90 mL of AgNO_3_ (1 mM) solution. The solution was then held for 24 h.

### 2.3. UV–Vis Spectroscopy

Synthesized *Camellia sinensis* silver nanoparticles (CS-AgNPs) were scanned by UV–Vis spectrophotometer (Lambda 25 spectrophotometer, Perkin-Elmer, Waltham, MA, USA) at the wavelength of 250–900 nm at different time intervals.

Scanning electron Microscopy (SEM) Transmission electron microscopy (TEM).

To better analyze the size and morphology of green synthesized *Camellia sinensis* silver nanoparticles (CS-AgNPs), electron microscopic techniques were used, viz. SEM (JSM 6510 LV, Peabody, MA, USA) and TEM (Jeol 2100, Tokyo, Japan). Briefly, a drop of the green synthesized nanoparticles was placed on the copper grid, and the sample was analyzed as described by Ali et al. [35].

### 2.4. X-ray Diffraction (XRD)

The crystalline or the amorphous nature of TG-AuNPs were determined using XRD (Rigaku, Pittsburg, PA, USA) with a scanning 2 theta angle from 5 to 80° at 40 KeV.

### 2.5. Tested Microorganisms

The bacterial and fungal isolates of *Escherichia coli*, Staphylococcus aureus, Klebsiella pneumoniae, Candida albicans, Candida glabrata, Candida dubliniensis, and Candida parapsilosis were obtained from the Department of Microbiology, J. N Medical College and Hospital, Aligarh Muslim University, India.

### 2.6. Evaluation of Minimum Inhibitory Concentration (MIC) of CS-AgNPs

The minimum inhibitory concentration of CS-AgNPs against microorganisms was determined using the broth dilution method [35,36]. Briefly, bacterial and fungal cultures were allowed to grow on nutrient agar a (NA)/Sabouraud dextrose agar (SDA) plate overnight at 37 °C and 28 °C, respectively. The colony was then picked from the overnight grown culture and inoculated into the nutrient broth/Saboraud dextrose broth and incubated at 37/28 °C for 5–6 h in a rotary shaking incubator. The CS-AgNPs were serially diluted with varying concentration of nanoparticles, and bacterial/fungal cultures were added [35].

### 2.7. Well Diffusion Assay

Initial assessment of antimicrobial efficacy of nanoparticles was performed using an agar diffusion assay [37,38]. Briefly, NA and SDA plates were prepared and wells were punched in the NA/SDA plates. The bases of the wells were sealed with soft agar. Varying concentrations of CS-AgNPs were poured in the wells of the NA/SDA plates, with the distilled water as a control.

### 2.8. Interaction of CS-AgNPs with Candida albicans

TEM (JEOL2100) was used to analyze the interaction between the CS-AgNPs and Candidal cells. Briefly, Sabouraud dextrose broth was inoculated with *Candida albicans* and incubated at 28 °C for overnight. Then, the overnight grown culture of 10^6^ CFU/mL was added to the SD broth containing 500 µg/mL of CS-AgNPs and further incubated at 28 °C for 12–18 h. After the incubation, the cells were washed with phosphate buffer saline (PBS) and fixed with 2.5% glutaraldehyde for 24 h. After fixation, the cells were again washed with PBS and dehydrated using alcohol. After dehydration, the cells were stored in PBS. A drop of suspension in PBS was placed on a copper grid and dried for viewing [37].

### 2.9. CLSM

The antibiofilm nature of silver nanoparticles was assessed using the CLSM technique. Briefly, 4 mL (BHI + 5% sucrose) were poured in 12-well microtiter plates, along with glass coverslips over each well. Overnight grown *C. albicans* were inoculated in the wells, along with 500 µg/mL of CS-AgNPs, and incubated at 28 °C for 24 h. After the incubation, the glass coverslips were washed with PBS and stained with Concanavalin A Fluorescein isothiocyanate (Con A-FITC), as per the protocol described by Ali et al. [39]. The effect of nanoparticles on biofilm was examined by confocal microscopy.

## 3. Result

### 3.1. Characterization of Silver Nanoparticles

**Figure 1:**
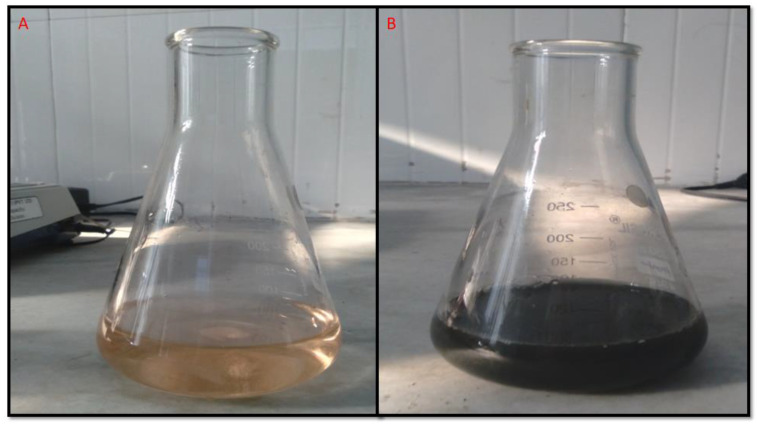
Representation of (**A**). silver nitrate mixed with plant extract soon after mixing; (**B**). silver nitrate with plant extract after 24 h.

Figure 1A represents the plant extract along with 1 mM AgNO_3_ soon after mixing, whereas Figure 1B represent the formation of nanoparticles detected by the change in color after 24 h of mixing of the plant extract with 1 mM AgNO_3_. The color change is associated with the excitation of surface plasmon resonance (SPR)

**Figure 2:**
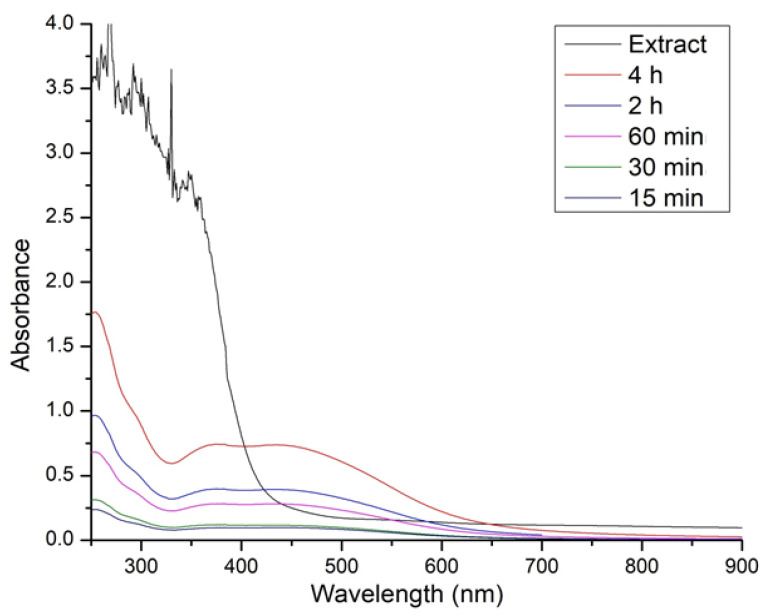
Representative of UV-Vis of silver nanoparticles at different time intervals.

The UV visible spectrum of CS-AgNPs at different time intervals, along with the plant extract, are shown below. Figure 2 shows the peak between 450–500 nm.

**Figure 3:**
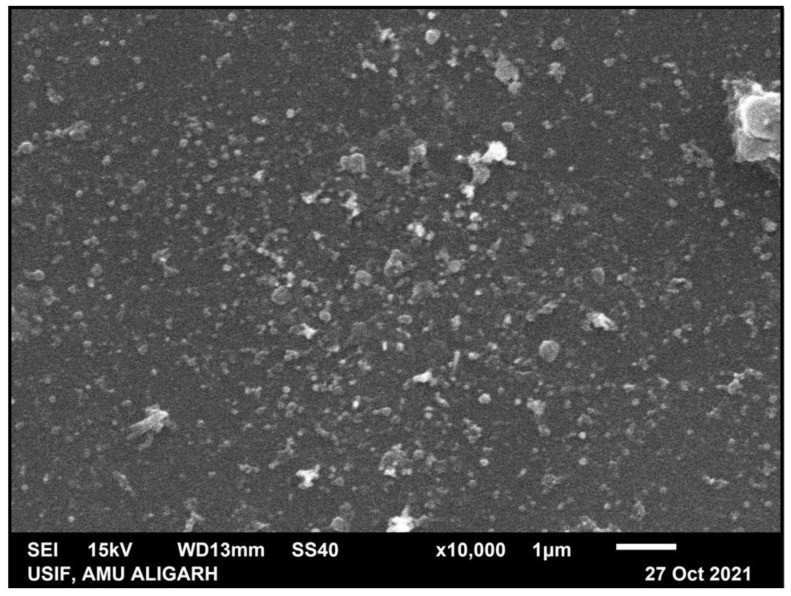
Representation of scanning electron microscopy (SEM) of CS-AgNPs.

**Figure 4:**
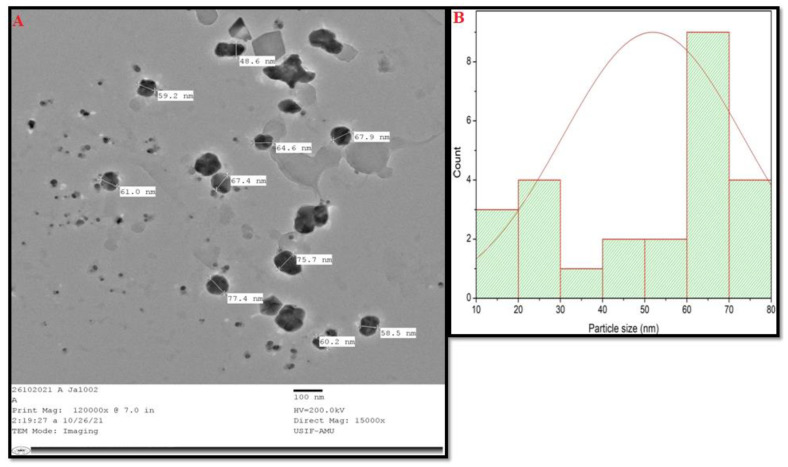
(**A**) Representation of transmission electron microscopy (TEM) of CS-AgNPs. (**B**) Particle size distribution of nanoparticles.

Figure 3 is indicative of SEM, which shows that nanoparticles have not formed, but are clumped, and neither aggregated nor uniformly distributed, whereas the TEM image is indicative of the size of nanoparticles, which was 52 nm (average size). TEM also shows that the nanoparticles are of varying shapes and sizes. Figure 4b represent the particle size distribution of nanoparticles via a histogram.

**Figure 5:**
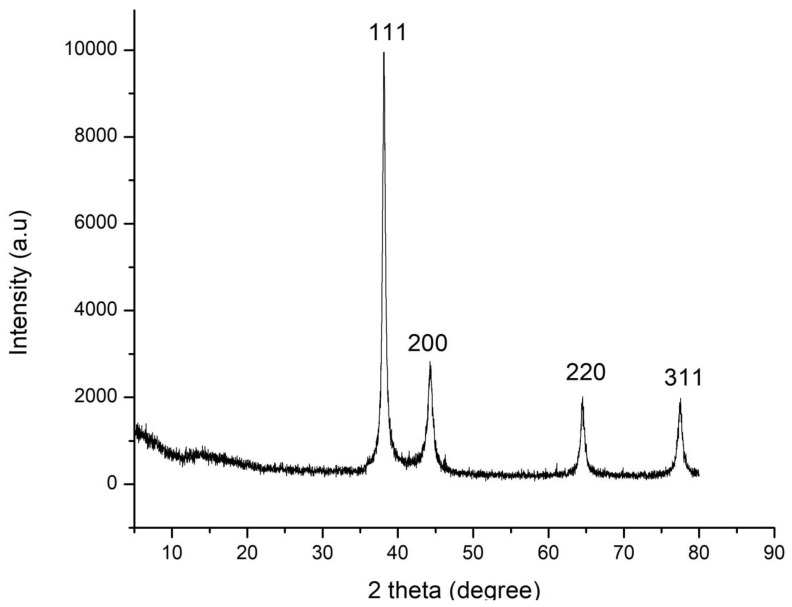
Representation of XRD.

The XRD analysis confirmed the crystalline nature of the nanoparticles. The diffraction peaks at 38.2°, 44.36, 64.48, and 77.4 related to 111, 200, 220, and 311 facets of the face centered cubic (FCC) crystal lattice, corresponded to silver (JCPDS card no 04-0783) (Figure 5).

The MIC of CS-AgNPs against bacterial isolates varied from 31.25 to 62.5 µg/mL, whereas for fungal isolates, MIC varied from 125 to 250 µg/mL. *E. coli* and *S. auerus* showed an MIC of 31.25 µg/mL, whereas *K. pneumoniae* showed an MIC of 62.5 µg/mL. Similarly *C. albicans* and *C. tropicalis* showed an MIC of 125 µg/mL, whereas *C. dubliniensis* and C. *parapsilosis* showed an MIC of 250 µg/mL.

### 3.2. Antimicrobial Activity of Nanoparticles

**Figure 6:**
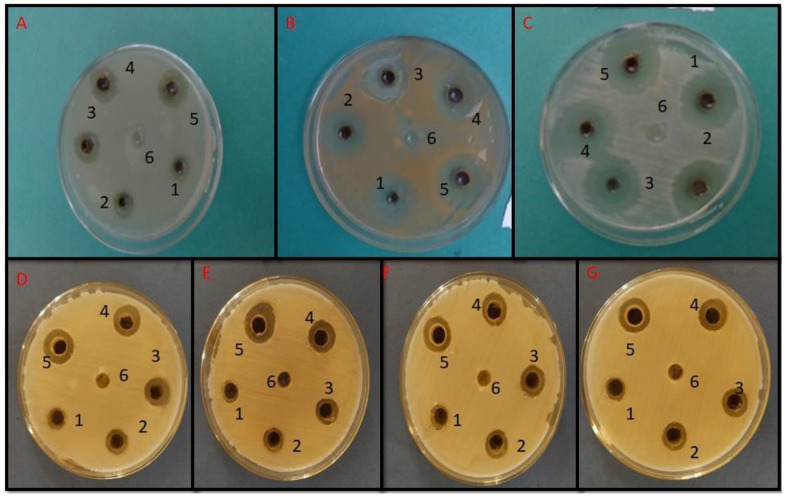
Well diffusion assay representative of (**A**) *K. pneumonia,* (**B**) *S*. *aureus,* (**C**) *E*. *coli,* (**D**) C. *albicans*, (**E**) *C*. *tropicalis,* (**F**) *C*. *dubliniensis,* (**G**) *C. parapsilosis.* 1 = 31.25 µg/mL, 2 = 62.5 µg/mL, 3 = 125 µg/mL, 4 = 250 µg/mL, 5 = 500 µg/mL, 6 = control (distilled water).

Figure 6 represents the antimicrobial activity of nanoparticles through well diffusion. The formation of zones is representative of the antimicrobial activity of CS-AgNPs. Figure 6A–C represents the antibacterial activity of CS-AgNPs against *K. pneumoniae, S. aureus,* and *E. coli*, whereas Figure 6D–G represents the antifungal activity of CS-AgNPs against *C albicans, C. tropicalis,* C *dubliniensis, C parapsilosis;* CS-AgNPs showed antibacterial activity at a lower concentration, whereas antifungal activity is seen at a higher concentration.

**Figure 7:**
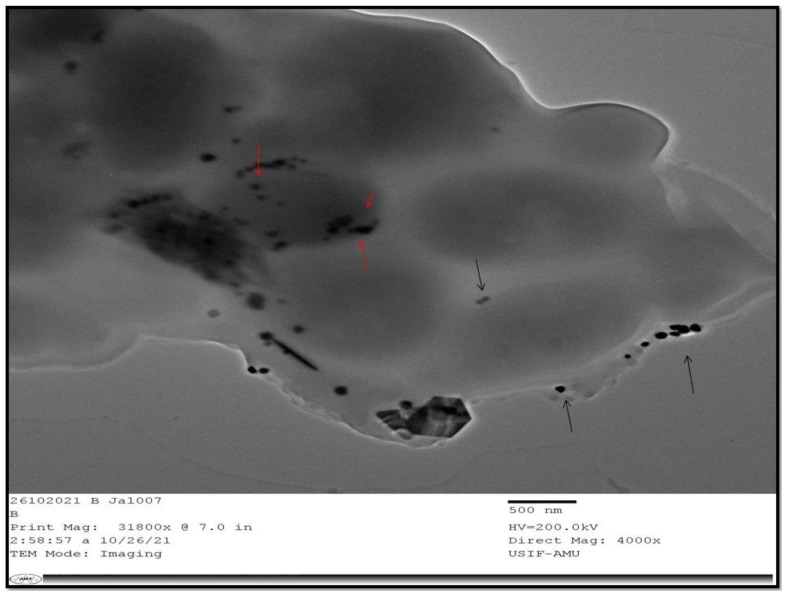
TEM image of *C. albicans* treated with CS-AgNPs at 500 µg/mL. Black arrows indicate CS-AgNPs at the surface, whereas red arrows indicate the internalization of CS-AgNPs.

The interaction of nanoparticles with the fungal cells is represented in Figure 7. The TEM image in Figure 7, is indicative of the internalization of the nanoparticles; the nanoparticles have also adhered onto the cell surface.

### 3.3. Antibiofilm Activity of Silver Nanoparticles through CLSM

**Figure 8:**
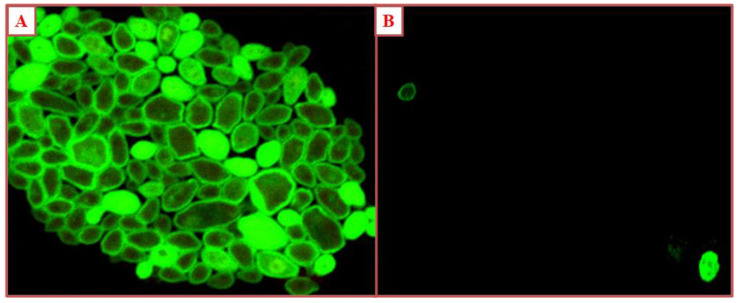
CLSM images of (**A**) C. *albicans* (Untreated); (**B**) 500 µg/mL of CS-AgNPs.

CLSM images of *C. albicans* after treatment with 500 µg/mL of silver nanoparticles showed that CS-AgNPs decrease biofilm formation. CS-AgNPs decrease the attachment of cells onto the surface, thereby decreasing biofilm formation (Figure 8).

## 4. Discussion

The present study describes the antibiofilm and antimicrobial nature of CS-AgNPs. Green synthesized silver nanoparticles (CS-AgNPs) were characterized using different techniques, including UV-Vis, SEM, TEM, and XRD. UV-Vis at different intervals (0–4 h), and it was concluded that the peak intensities were raised between 450–500 nm. The intensity was increased due to the surface plasmon resonance. The SPR is basically due to the presence of free electrons, which arise from the conduction and valence bands, since they are close to each other in metals [8,40]. The SPR gives the initial sign of the synthesis of nanoparticles. Our results are in agreement with the previous studies regarding UV-Vis of green synthesized nanoparticles after mixing plant extract with silver nitrate at different time intervals [25]. The characterization proved that nanoparticles were not clumped nor aggregated, and the average size was 52 nm. It has previously been shown that the antimicrobial activity in the metal nanoparticles is the function of the size of the nanoparticles [41]

Figure 4b represents the particle size distribution, which shows the presence of different nanoparticles, with variable size. Further, the XRD analysis also confirmed the crystalline nature of silver nanoparticles. The MIC of CS-AgNPs against bacteria was low compared with fungi. The antimicrobial activity of silver nanoparticles was further assessed using a well diffusion assay, and it was found that CS-AgNPs showed good antimicrobial activity, although zones were formed at a lower concentration for bacteria, whereas for fungi, a higher concentration of CS-AgNPs was required. This may be due to the fact that bacterial cells are prokaryotic in evolution, having a less complex structure; therefore, they could not resist the toxicity of silver nanoparticles, to a greater extent, whereas fungi, being eukaryotic in origin and with a complex structure, can resist the toxicity of silver nanoparticles to a greater extent than bacteria. [38,42]. Previous studies have shown that AgNPs decreased the bacterial populations from Bacteroides, Enterobacteriaceae, and Lactobacillus, whereas the Bifidobacterium group was favored [43]. It has also been shown that the association of silver nanoparticles with the plant extract, such as wild mushroom, had a synergistic antimicrobial activity against different pathogenic microorganisms, whereas the extract reduced the formation of biofilm [43]. Green tea contains polyphenols, which have important immunomodulatory and anti-inflammatory functions. In vitro studies have shown that these polyphenols helps in selective metabolization, accounting for 20% of the assimilation rate [44].

*Candida albicans* form a biofilm, which makes it adhere more strongly and compactly to the surface, developing resistance to antifungals [45]. The extracellular polymeric substance secreted by *C. albicans* prevents the diffusion of antifungal drugs into the cell [34]. The biofilm is itself a problem of great concern, which should be eradicated. Silver nanoparticles decreases the biofilm formation, which can be observed through CLSM images. The CLSM image shows that at 500 µg/mL of CS-AgNPs, a minimum number of cells remain attached on the surface, showing the reduction in biofilm formation. We are also of the opinion that CS-AgNPs arrest the release of exopolysaccharide, which does not allow Candidal cells to adhere onto the surface. Our results are in agreement with the previous studies of Ahamad et al. [46], which revealed the inhibition of *Candida albicans* biofilm using biogenic silver nanoparticles. Further, Figure 7 represents the interaction of nanoparticles with the fungal cells, and it is very clear that all the nanoparticles are not internalized in the *Candida albicans*; rather, few nanoparticles are found at the surface of the Candidal cells (black arrow), whereas some of the nanoparticles have internalized the Candidal cells (red arrow). These surface-attached and internalized nanoparticles have probably disturbed the usual mechanism of the Candidal cells; therefore, cell death has occurred. Our results are in agreement with the previous studies of Vazquez-Muñoz et al. [47], which presented the interaction of silver nanoparticles with Candidal cells and the accumulation of silver nanoparticles. Vazquez-Munoz et al. [47] concluded that silver nanoparticles accumulate outside the cell and released the silver ions, which induces cell death through the reduction process.

Hamida et al., [48] showed that silver nanoparticles synthesized from cyanobacteria, when interacted with *C. albicans,* degraded and shrank the cell wall and cell membrane. Similarly, Jalal et al., [49] also showed that *syzygium cumini*-mediated silver nanoparticles damaged the cell wall and cytoplasmic membrane of *C. albicans*.

## 5. Conclusions

The green synthesis method of silver nanoparticles is a cost effective and efficient method. In our study, we have considered *Camellia sinensis* (green tea) for silver nanoparticles synthesis. The synthesized silver nanoparticles (CS-AgNPs) were characterized using different techniques, viz. UV-Vis, SEM, TEM, and XRD. The silver nanoparticles showed good antibacterial and antifungal activity against clinical pathogens, as revealed by the well diffusion assay. The interaction of CS-AgNPs with the Candidal cells, as seen using TEM, showed the antimicrobial nature of silver nanoparticles. Through the CLSM analysis, it was also confirmed that CS-AgNPs possess strong anti-biofilm activity. Hence, it is concluded that green synthesized silver nanoparticles may be used against bacterial/fungal infection, but more research needs to be done on the molecular level to identify the effect of silver nanoparticles at the gene level.

## Data Availability

The study did not report any data.

## References

[1] Ahmed M.J., Murtaza G., Mehmood A., Bhatti T.M. (2015). Green synthesis of silver nanoparticles using leaves extract of *Skimmia laureola*: Characterization and antibacterial activity. Mater. Lett..

[2] Ali K., Dwivedi S., Azam A., Saquib Q., Al-Said M.S., Al-Khedhairy A., Musarrat J. (2016). *Aloe vera* extract functionalized zinc oxide nanoparticles as nanoantibiotics against multi-drug resistant clinical bacterial isolates. J. Colloid Interface Sci..

[3] Ambika S., Sundrarajan M. (2016). [EMIM] BF4 ionic liquid-mediated synthesis of TiO_2_ nanoparticles using *Vitex negundo Linn* extract and its antibacterial activity. J. Mol. Liquids..

[4] Ahmad S., Munir S., Zeb N., Ullah A., Khan B., Ali J., Bilal M., Omer M., Alamzeb M., Salman S.M. (2019). Green nanotechnology: A review on green synthesis of silver nanoparticles—An ecofriendly approach. Int. J. Nanomed..

[5] Shah M., Fawcett D., Sharma S., Tripathy S.K., Poinern G.E.J. (2015). Green synthesis of metallic nanoparticles via biological entities. Materials.

[6] Rafique M., Sadaf I., Rafique M.S., Tahir M.B. (2017). A review on green synthesis of silver nanoparticles and their applications. Artif. Cells Nanomed. Biotechnol..

[7] Jyoti K., Baunthiyal M., Singh A. (2016). Characterization of silver nanoparticles synthesized using *Urtica dioica Linn*. leaves and their synergistic effects with antibiotics. J. Radiat. Res. Appl. Sci..

[8] Dada A.O., Adekola F.A., Adeyemi O.S., Bello M.O., Adetunji C.O., Awakan O.J., Grace F.A.A. (2018). Exploring the effect of operational factors and characterization imperative to the synthesis of silver nanoparticles. Silver Nanoparticles—Fabrication, Characterization and Applications.

[9] Abou El-Nour K.M.M., Eftaiha A., Al-Warthan A., Ammar R.A.A. (2010). Synthesis and applications of silver nanoparticles. Arab. J. Chem..

[10] Prabu H.J., Johnson I. (2015). Antibacterial activity of silver nanoparticles synthesized from plant leaf extract of *Cycas circinalis*, *Ficus amplissima*, *Commelina benghalensis* and *Lippia nodiflora* leaves. J. Chem. Pharm. Res..

[11] Siddiqi K.S., Husen A., Rao R.A.K. (2018). A review on biosynthesis of silver nanoparticles and their biocidal properties. J. Nanobiotechnol..

[12] Kambale E.K., Nkanga C.I., Mutonkole B.P.I., Bapolisi A.M., Tassa D.O., Liesse J.M., Krause R.W.M., Memvanga P.B. (2020). Green synthesis of antimicrobial silver nanoparticles using aqueous leaf extracts from three Congolese plant species (*Brillantaisia patula*, *Crossopteryx febrifuga* and *Senna siamea*). Heliyon.

[13] Vijayan R., Joseph S., Mathew B. (2018). Green synthesis, characterization and applications of noble metal nanoparticles using *Myxopyrum serratulum* A. W. Hill leaf extract. Bionanoscience.

[14] Liao C., Li Y., Tjong S.C. (2019). Bactericidal and cytotoxic properties of silver nanoparticles. Int. J. Mol. Sci..

[15] Wang Y., Maksimuk S., Shen R., Yang H. (2007). Synthesis of iron oxide nanoparticles using freshly- made or recycled imidazolium-based ionic liquid. Green Chem..

[16] Horwat D., Zakharov D.I., Endrino J.L., Soldera F., Anders A., Migot S., Karoum R., Vernous Ph., Pierson J.F. (2011). Chemistry, phase formation, and catalytic activity of thin palladium-containing oxide films synthesized by plasma-assisted physical vapor deposition. Surf. Coat. Technol..

[17] Devi H.S., Boda M.A., Shah M.A., Parveen S., Wani A.H. (2019). Green synthesis of iron oxide nanoparticles using *Platanus orientalis* leaf extract for antifungal activity. Green Process. Synth..

[18] Alsammarraie F.K., Wang W., Zhou P., Mustapha A., Lin M. (2018). Green synthesis of silver nanoparticles using turmeric extracts and investigation of their antibacterial activities. Colloids Surf. B Biointerfaces.

[19] Kataria N., Garg V.K. (2018). Green synthesis of Fe_3_O_4_ nanoparticles loaded sawdust carbon for cadmium (II) removal from water: Regeneration and mechanism. Chemosphere.

[20] Baruwati B., Polshettiwar V., Varma R.S. (2009). Glutathione promoted expeditious green synthesis of silver nanoparticles in water using microwaves. Green Chem..

[21] Ahmed S., Ikram S. (2015). Silver nanoparticles: One pot green synthesis using Terminalia arjuna extract for biological application. J. Nanomed. Nanotechnol..

[22] Tashi T., Gupta N.V., Mbuya V.B. (2016). Silver nanoparticles: Synthesis, mechanism of antimicrobial action, characterization, medical applications, and toxicity effects. J. Chem. Pharm. Res..

[23] Natsuki J., Natsuki T., Hashimoto Y. (2015). A review of silver nanoparticles: Synthesis methods, properties and applications. Int. J. Mater. Sci. Appl..

[24] Yaqoob A.A., Umar K., Ibrahim M.N.M. (2020). Silver nanoparticles: Various methods of synthesis, size affecting factors and their potential applications—A review. Appl. Nanosci..

[25] Dada A.O., Inyinbor A.A., Idu E.I., Bello O.M., Oluyori A.P., Adelani-Akande T.A., Dada O. (2018). Effect of operational parameters, characterization and antibacterial studies of green synthesis of silver nanoparticles using *Tithonia diversifolia*. PeerJ.

[26] Dada A.O., Adekola F.A., Dada F.E., Adelani-Akande A.T., Bello M.O., Okonkwo C.R., Inyinbor A.A., Oluyori A.P., Olayanju A., Ajanaku K.O. (2019). Silver nanoparticle synthesis by *Acalypha wilkesiana* extract: Phytochemical screening, characterization, influence of operational parameters, and preliminary antibacterial testing. Heliyon.

[27] Ali S.G., Ansari M.A., Khan H.M., Jalal M., Mahdi A.A., Cameotra S.S. (2018). Antibacterial and antibiofilm potential of green synthesized silver nanoparticles against imipenem resistant clinical isolates of *P. aeruginosa*. Bionanoscience.

[28] Femi-Adepoju A.G., Dada A.O., Otun K.O., Adepoju A.O., Fatoba O.P. (2019). Green synthesis of silver nanoparticles using terrestrial fern (*Gleichenia pectinate* (Wild.) C. Presl.): Characterization and antimicrobial studies. Heliyon.

[29] Bello O.M., Oguntoye O.S., Dada A.O., Bello O.E., Ali T., Alhaji A.A., Adeniyi O. (2019). Phytobiological facilitated production of silver nanoparticles from selected non-cultivated vegetables in Nigeria and their biological potential. Turk. J. Pharm. Sci..

[30] Yousaf H., Mehmood A., Ahmad K.S., Raffi M. (2020). Green synthesis of silver nanoparticles and their applications as an alternative antibacterial and antioxidant agent. Mater. Sci. Eng. C.

[31] Dominguez E.G., Zarnowski R., Choy H.L., Zhao M., Sanchez H., Nett J.E., Andes D.R. (2019). Conserved role for biofilm matrix polysaccharides in *Candida auris* drug resistance. MSphere.

[32] Kean R., Delaney C., Sherry L., Borman A., Johnson E.M., Richardson M.D., Rautemma-Richardson R., Williams C., Ramage G. (2018). Transcriptome assembly and profiling of *Candida auris* reveals novel insights into biofilm-mediated resistance. MSphere.

[33] Douglas L.J. (2003). *Candida* biofilms and their role in infection. Trends Microbiol..

[34] Seneviratne C.J., Wang Y., Jin L., Abiko Y., Samaranayake L.P. (2008). *Candida albicans* biofilm formation is associated with increased anti-oxidative capacities. Proteomics.

[35] Ali S.G., Ansari M.A., Alzohairy M.A., Alomary M.N., Jalal M., AlYahya S., Asiri S.M.M., Khan H.M. (2020). Effect of Biosynthesized ZnO Nanoparticles on Multi-Drug Resistant Pseudomonas Aeruginosa. Antibiotics..

[36] Ansari M.A., Khan H.M., Alzohairy M.A., Jalal M., Ali S.G., Pal R., Musarrat J. (2014). Green synthesis of Al_2_O_3_ nanoparticles and their bactericidal potential against clinical isolates of multi -drug resistant *Pseudomonas aeruginosa*. World J. Microbiol. Biotechnol..

[37] Jalal M., Ansari M.A., Shukla A.K., Ali S.G., Khan H.M., Pal R., Alam J., Cameotra S.S. (2016). Green synthesis and antifungal activity of Al_2_O_3_ NPs against fluconazole resistant *Candida* spp. isolated from a tertiary care hospital. RSC Adv..

[38] Jalal M., Ansari M.A., Alzohairy M.A., Ali S.G., Khan H.M., Almatroudi A., Raees K. (2018). Biosynthesis of Silver Nanoparticles from Oropharyngeal *Candida glabrata* Isolates and Their Antimicrobial Activity against Clinical Strains of Bacteria and Fungi. Nanomaterials..

[39] Ali S.G., Ansari M.A., Khan H.M., Jalal M., Mahdi A.A., Cameotra S.S. (2017). *Crataeva nurvala* nanoparticles inhibit virulence factors and biofilm formation in clinical isolates of *Pseudomonas aeruginosa*. J. Basic Microbiol..

[40] Anandalakshmi K., Venugobal J., Ramasamy V. (2016). Characterization of silver nanoparticles by green synthesis method using *Pedalium murex* leaf extract and their antibacterial activity. Appl. Nanosci..

[41] Tippayawat P., Phromviyo N., Boueroy P., Chompoosor A. (2016). Green synthesis of silver nanoparticles in aloe vera plant extract prepared by a hydrothermal method and their synergistic antibacterial activity. PeerJ.

[42] Panacek A., Kolar M., Vecerova R., Prucek R., Soukupova J., Krystof V., Hamal P., Zboril R., Kvitek L. (2009). Antifungal activity of silver nanoparticles against *Candida* spp.. Biomaterials.

[43] Vamanu E., Ene M., Bita B., Ionescu C., Craciun L., Sarbu I. (2018). In Vitro Human Microbiota Response to Exposure to Silver Nanoparticles Biosynthesized with Mushroom Extract. Nutrients.

[44] Vamanu E., Gatea F. (2020). Correlations between Microbiota Bioactivity and Bioavailability of Functional Compounds: A Mini-Review. Biomedicines.

[45] Forsberg K., Woodworth K., Walters M., Berkow E.L., Jackson B., Chiller T., Vallabhaneni S. (2019). *Candida auris*: The recent emergence of a multidrug-resistant fungal pathogen. Med. Mycol..

[46] Ahamad I., Fareha B., Anwer R., Srivastava P., Kumar R., Fatma T. (2022). Antibiofilm activities of Biogenic Silver nanoparticles against *Candida albicans*. Front. Microbiol..

[47] Vazquez-Muñoz R., Avalos-Borja M., Castro-Longoria E. (2014). Ultrastructural analysis of *Candida albicans* when exposed to silver nanoparticles. PLoS ONE.

[48] Hamida R.S., Ali M.A., Goda D.A., Redhwan A. (2021). Anticandidal potential of two Cyanobacteria-synthesized silver nanoparticles: Effects on growth, cell morphology, and key virulence attributes of *Candida albicans*. Pharmaceutics.

[49] Jalal M., Ansari M.A., Alzohairy M.A., Ali S.G., Khan H.M., Almatroudi A., Siddiqui M.I. (2019). Anticandidal activity of biosynthesized silver nanoparticles: Effect on growth, cell morphology, and key virulence attributes of *Candida* species. Int. J. Nanomed..

